# Renewable energy from secondary wood products contributes to local green development: the case of small-scale privately owned forests in Ciamis Regency, Indonesia

**DOI:** 10.1186/s13705-023-00383-7

**Published:** 2023-02-14

**Authors:** Mohamad Siarudin, San Afri Awang, Ronggo Sadono, Priyono Suryanto

**Affiliations:** 1 National Research and Innovation Agency (BRIN), Jakarta, Indonesia; 2 Department of Forest Management, Faculty of Forestry, Universitas Gadjah Mada, Yogyakarta, Indonesia; 3 Department of Silviculture, Faculty of Forestry, Universitas Gadjah Mada, Yogyakarta, Indonesia

**Keywords:** Biomaterial, Chain of production, Local wisdom, Logging, Sawmill

## Abstract

**Background:**

Wood biomass from forests is a renewable energy source that has the potential to support global green development. However, the process of traditional firewood extraction and its contribution to the energy supply varies and is usually underrecognized, especially in the local context. Therefore, this study aimed to describe the traditional use and estimate the supply and demand for wood bioenergy from small-scale privately owned forests (SSPF) in Ciamis Regency, West Java Province, Indonesia.

**Methods:**

The sample location was determined in 3 subdistricts, namely, Sukamantri, Ciamis, and Banjaranyar, which represent the northern, central, and southern regions, respectively. The data were collected through observations on stands, interviews with respondents, key informants from users and business actors of wood in the SSPF, and observations on the processes taking place in the flow and use of biomaterials and firewoods.

**Results and conclusion:**

Firewood is a secondary product that cannot be separated from the main products along SSPF’s chain of production based on the integration of raw material sources, linkages between actors, and volume sharing. The local knowledge and wisdom entanglements included the identification of the type and quality of firewood, distribution of resource allocation for household and industrial needs, and sharing of firewood for household needs. Although wood biomass contributes to the fulfillment of households and industrial needs of 8.51 million m^3^, there are indications of an imbalance between the potential supply and the demand for firewood due to its high intensity of use in industries. Therefore, multistakeholder and cross-regional support are needed to achieve sustainable SSPF management and fulfill the self-sufficiency of wood energy.

**Supplementary Information:**

The online version contains supplementary material available at 10.1186/s13705-023-00383-7.

## Background

Not only the limitations of fossil energy sources but also targets to mitigate climate change have led to a global commitment to the use of renewable energy sources such as biomass energy. The energy sector is among the sustainable development goals (SDGs) that target a significant increase in the share of renewable energy in the energy mix [1]. Currently, approximately 81% of the global economy is based on the fossil fuels, in addition to 5% on nuclear energy and 14% on renewable energy [2], where the use of renewable energy increases in response to population growth [3]. From 1990 to 2018, the global use of renewable energy increased by 2.1% per year, with a consumption rate of 64.2 exajoules in 2018 [4, 5]. A previous study also reported that the usage of renewable energy is more resilient during the COVID-19 pandemic than other energy sources [4].

Among the global renewable energy sources, biomass contributes approximately 70% [2]. In total, 11.9 B tons of biomass were supplied from agriculture (61%) and forestry (39%), of which the forest woody biomass for bioenergy accounted for 23% while that for biomaterial was only 8% [2]. There is an increase in the demand for using bioenergy because the low carbon economy has become a global commitment [2, 5]. It is estimated that the use of wood for bioenergy in 2050 ranges from 0 to 23 Gm^3^/year [6]. From the power sector, the use of biomass could supply 3000 Terawatt hours (TWH) of electricity and contribute to a 10–30 Gt emission reduction by 2050 [7]. The energy products derived from biomass conversion can be in the form of heat or electricity, as well as biofuel inclusive of, e.g., wood pellets, split logs, and wood charcoal [8].

In Indonesia, the renewable energy mix is reported to have reached 8.6% by 2019. In future scenarios, the domestic fossil energy supply in Indonesia will only meet 75% of 2030 needs and 28% of 2045 needs. The Indonesian government is targeting 23% of the new and renewable energy mix by 2025 to meet this energy scarcity [9]. The forestry sector itself is one of the sectors targeted to contribute to the fulfillment of renewable energy, particularly biomass energy, through the development of energy plantations. The Indonesian Ministry of Environment and Forestry targets the development of an energy plantation forest (EPF) of 800 thousand ha by 2025 [10]. Indonesia is predicted to generate 146.7 million tons of biomass per year, which equates to approximately 470 GJ/year [11]. The woody biomass of Indonesian forests, including log cutting residues, sawn timber, and wood industries, is estimated at 15.8 million ton/year, with an energy potential of 141.5 million GJ/year [12].

Forests are the largest source of woody vegetation (trees) on Earth and can be a source of biomass for energy. For the past thousands of years, humans have used tree biomass as a source of energy for cooking, heating, and industrial processes [13, 14]. Before the twentieth century, wood was among the global main sources of energy, but it has been partially replaced by coal, oil, and natural gas over the last century [15]. Some of the advantages of wood as bioenergy are its renewable potential through tree planting and by nature, the fact that human management provides additional value to wood waste, creates local jobs, generates intermediate income [16–18], and lower environmental impacts than fossil energy [19], so it can be an effective tool for the green economy by mitigating the impact of greenhouse gases [1, 20]. The use of biomass energy in the form of firewood also has an impact on air pollution emissions [21] and CO_2_ emissions in the combustion process [22, 23]. This is especially the case with the traditional use of firewood with low efficiency [21, 24]. The negative impact of using bioenergy can also occur due to taking dead wood in forestland instead of leaving it [24, 25]. However, the use of firewood has lower net CO_2_ emissions than nonrenewable energy use [24]. Although CO_2_ emissions are unavoidable in the use of firewood, sustainable forest management can offset the CO_2_ emissions of fossil energy use by saving less than 10% of the net increment [23]. In addition, biomass energy consumption may increase the ecological footprint but decrease CO_2_ emissions by 0.02–0.09%, at least according to the case of five biomass energy consuming countries (Brazil, China, Germany, India, and the United States [1].

The global demand to promote sustainable renewable energy development requires a response with real action and the support of scientific justification. Because excessive use of wood energy will increase the pressure on natural resources, it is necessary to develop strategies for achieving sustainable forest management while promoting the consumption of clean energy technologies [26]. Several previous studies have been conducted to evaluate the nexus of wood energy uses and the sustainability of forest resources, e.g., [27]; to evaluate the carbon footprint related to the woody bioenergy production process at the stage of silviculture and harvesting, e.g., [28]; or along supply chains, e.g., [28]. Each region has geographic, biophysical, and sociodemographic characteristics that can have an impact on the tradition of using firewood as an energy source [29]. Therefore, paying attention to local perspectives to meet global demands will be one of the keys to developing the use of renewable energy. This study aimed to describe the traditional usage and estimate the supply and demand of energy wood sourced from the SSPF in the Ciamis Regency for households and industry. The Ciamis Regency was chosen as the study location because this area represents one of the large SSPFs in West Java Province [30, 31]. A study in 2015 showed that rural communities in Ciamis Regency still use firewood from the SSPF [32]. A report by the Local Government of Ciamis Regency indicates that there were more than 1800 micro, small- and medium-scale industries that use firewood from the SSPF as an energy source in 2020 [33]. Initial observations showed that the use of firewood on a household and industrial scale still persists as an energy source in this region. However, the balance between supply and demand for firewood has not yet been taken into account. Population development and the number of industries were thought to lead to an imbalance between the supply and demand for firewood. The results were expected to show the important value of the SSPF based on its contribution to the supply of wood energy for households and industries. In addition, it can be used as a reference for the development of SSPF as a source of renewable energy.

## Methods

### Study location

**Fig. 1:**
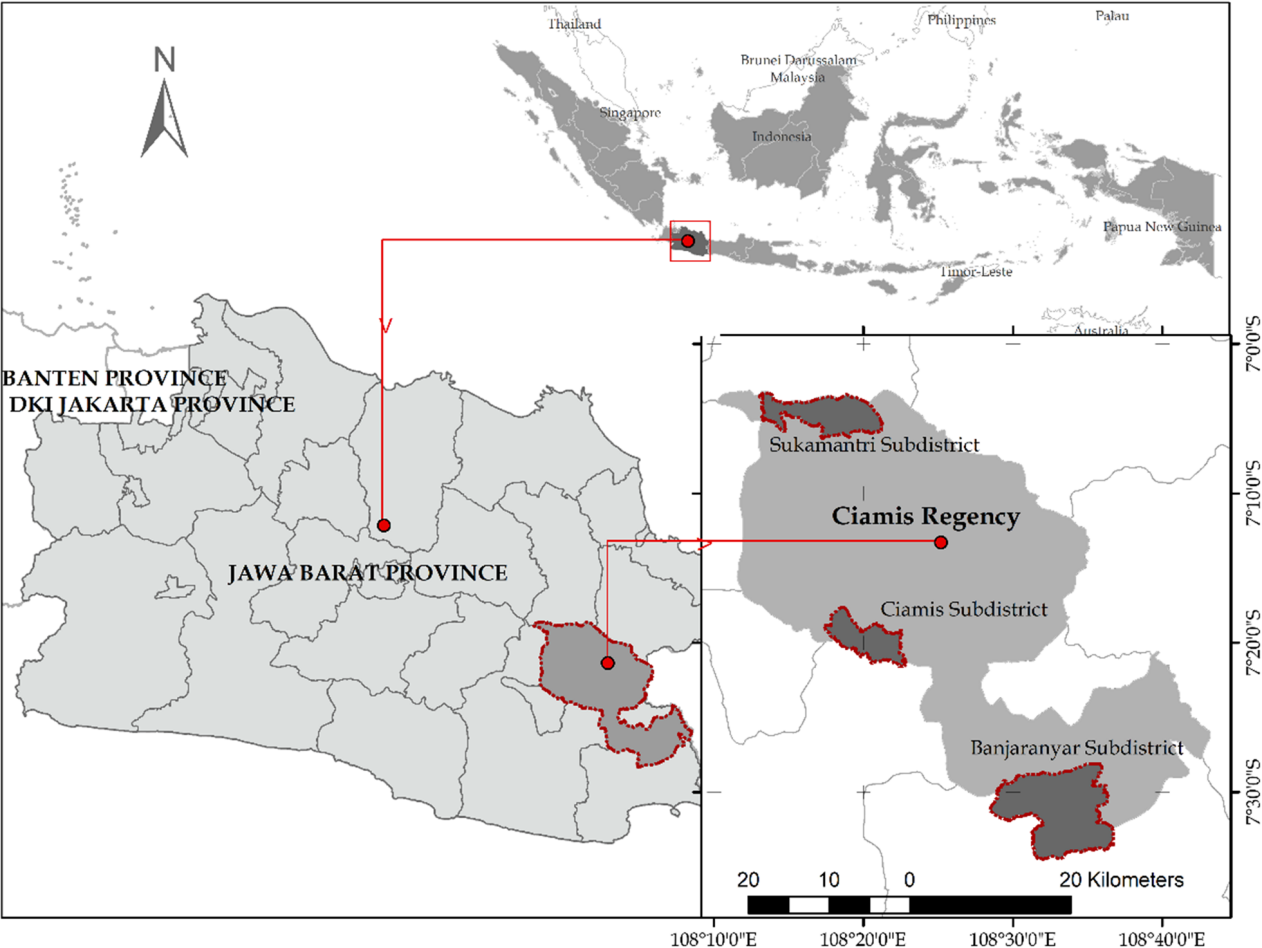
Map of the Ciamis Regency and three subdistricts of sample locations

This study was conducted in the Ciamis Regency of West Java Province, Indonesia (Fig. 1). The regency spans 1536.84 km^2^, with elevations ranging from 27 to 842 m above sea level (masl). The regency has an average monthly temperature of 19.54–20.32 °C, 82.85–85.95% humidity, and an average rainfall of 2110.16 mm/year between 2002 and 2012 [34]. The total area of small-scale privately owned forest (SSPF) reaches 33% of the total area of Ciamis Regency [35].

The sample location was carried out in 3 subdistricts, which represent the geographical region and the altitude strata (Table 1). The altitude strata were selected based on the subdistrict with the highest, lowest, and between the two based on the data from the Central Bureau of Statistics of Ciamis Regency. Sukamantri Subdistrict in the northern region represents the highest plain, Ciamis Subdistrict in the middle area represents the intermediate plains, and Banjaranyar Subdistrict in the south region represents the lowlands [34]. The southern part is a steep plateau with slopes between 15 and 40%, while the middle and southern parts are lowlands with 0–15% slopes [39]. The selection of strata was based on the assumption that altitude affects biophysical conditions, plant species suitability, community culture, and road access, with the potential to influence forest characteristics [40].

**Table 1:** Some key data of the study location

No.	Key data	Sukamantri Subdistrict	Ciamis Subdistrict	Banjaranyar Subdistrict	Ciamis Regency
1	Total area (ha)	4788	3277	9458	153,684
2	SSPF area (ha)	2123	391	6750	50,192
3	Number of residents (persons)	29,692	109,839	47,016	1,430,262
4	Population density (persons/km^2^)	620	3352	496	931
5	Altitude (masl)	775	202	48	27–842

Source: [34–38]

*Masl* meters above sea level, *SSPF* small-scale privately owned forest

SSPF in Ciamis Regency is a tree-based land-use system that is implemented on privately owned lands. In 2019, it was reported that the area of SSPF in Ciamis was 50,192 ha, with a roundwood production of 194,395 m^3^ [41, 42]. The existence of community forests in the Ciamis Regency also promotes local economic activities, as shown by the increase in the wood processing industries, which reached 477 units and employed 6474 workers, with an investment value of 54.1 billion Indonesian rupiah (IDR) in 2019 (1 USD =  ± 15,000 IDR) [33]. The SSFP in Ciamis Regency is also one of the suppliers of wood energy for households and industries sourced from dead branches, twigs in the forest, and residues from logging activities and sawmill industries.

### Data collection

The data collected in this study are intended to obtain information regarding the general overview of the SSPF, the chain of production and wood material flow, and the potential firewood supply and demand (Table 2). Data collection was started with initial field surveys at the sites to obtain an overview of the SSPF stands, while the general cropping patterns and dominant tree species were observed. Discussions with key informants, including 2 local government officials, 4 forestry extension agents, and 3 forest farmer group administrators, confirmed the observations on cropping patterns and dominant species. In addition, dominant tree species were tracked at the logging activity measurements and confirmed by secondary data from the Forestry Office report.

**Table 2:** Data source and data collection methods

No.	Data	Source of data	Data collection method
1	General overview of SSPF		
	• Physical condition of SSPF	Field	Field survey
	• Management practice	9 Key informants	Interview
	• Major tree species	Local government report	Desk study
		Field	Observation
2	Chain of production and wood material flow	19 Loggers	Observation, interview
		14 Wood waste buyers	Observation, interview
		14 Sawmill industries	Observation, interview
		30 Key informants	Interview
3	Firewood potential supply		
	• Wood waste volume in logging activities	19 Loggers	Interview
	• Wood waste volume in sawmill industries	14 Sawmill industries	Interview
		8 Firewood collectors	Interview
	• Annual roundwood production	Local government report	Desk study
	• Number of sawmill industries	Local government report	Desk study
4	Firewood demand		
	• Annual firewood consumption per household; reason for energy source usage	140 Heads of households	Interview
	• Annual firewood consumption per industry	31 Industries 8 Firewood collectors	Interview
	• Number of firewood-user industries	Local government report	Desk study
	• Number of residents	Local government report	Desk study

In this study, the bioenergy that is investigated is the woody materials used as firewood in the community and industry. The observations covered the SSPF stands, logging activities, and sawmill industries. In addition, the secondary product used in this study refers to a byproduct, a coproduct, or residue [43] produced along the production chain of wood forest products. This distinguishes the primary products of wood biomaterials such as merchantable roundwoods, sawn timbers, veneers, and others.

The observation of the firewood covered the stands of deadwood from forest vegetation, specifically the dead twigs and branches, stumps, midribs, or other parts commonly collected by the community, logging waste, and biomass waste such as slabs, sideboard, or sawdust from the wood processing industry (see Additional file 1: Fig. S1). The logging waste itself can be derived into twigs with a diameter of less than 5 cm and branch or small-diameter roundwood with a diameter of 5–10 cm.

Observations on the production chain of wood and firewood-based forest products were carried out by evaluating and inspecting the business process of the chains to obtain an overview of the actors and flows of wood materials. The actors involved in the production chain of timber forest products and industries that use firewood were identified by key informants, especially the Forestry Service Branch (4 people), Cooperative and Small and Medium Industry Service (2 people), Forestry Extension Officers (4 people), Local Government Officers at the subdistrict and village levels (15 people), and forestry business actors (5 people). Subsequently, interviews were conducted with informants from the actors to obtain data on the volume of roundwood processing, residues in logging and sawing, and the relationship between actors and material flows. In addition, 150 households (50 respondents per subdistrict) were interviewed with a questionnaire tool to obtain information on energy sources used for household purposes and estimates of firewood consumption. Those 150 respondents were chosen by considering their availability to interview and considering the distribution of their location after discussion with the local community leaders of subdistrict and village levels. The 50 respondents per subdistrict were based on the need for a normal distribution, which requires a minimum of 30 sample sizes [44]. This total number of respondents met the minimum requirements of 100 people to be able to represent a population > 100,000 with a 10% precision level (*e*), where the confidence level is 95% and the *P* value is 0.5 [45]. The final number of respondents included in the data analysis was 140 (46 from Ciamis Subdistrict, 48 from Banjaranyar Subdistrict, and 46 from Sukamantri Subdistrict) after data cleaning on inaccurate or incomplete data.

The potential of firewood for the industry was observed by measuring wood waste volume both in logging activities and sawmill industries. This was conducted through interviews with 19 loggers and the observation of 3–5 logging events per logger with a total of 68 logging events. Interviews were also conducted with 8 firewood collectors and 14 sawmill industry representatives. The data on annual firewood consumption per industry were obtained through interviews with 3 medium-scale industries and 28 small- and microscale industry agents. The industrial scale is based on the criteria of Small Enterprises Law No. 9 of 1995, and the type of industry is based on the report of Industrial Potential of Ciamis Regency Year 2020 [33].

Respondents for loggers, firewood collectors, and sawmill industries were randomly selected in 3 sample subdistricts. Given that there are no official data on the total number of loggers and collectors in the area, the group size was estimated through interviews with key informants of 3 loggers and 3 firewood collectors. The number of samples was determined to be at least 50% of the estimated group in the research location. In addition, industry respondents using firewood are quite diverse, including tofu, copra, snacks, palm/coconut sugar, poultry farm, brick, and cracker industries. For each type of industry, at least 3 respondents were randomly selected for interviews. To improve the accuracy of the data from these respondents, validation was carried out through observation and interviews with key informants of 8 firewood collectors who supply the industry.

### Data analysis

This study employed qualitative and quantitative approaches. Qualitative analysis was used to describe the general condition of SSPF stands and their management practices, as well as the interrelationships among actors and the processes that occur along the production chains. Quantitative analyses were used in the estimation of the potential supply and consumption (demand) of firewood for families and industry. Several calculations were applied to the sample data to obtain the ratio of the wood waste volume to the main product volume both in the logging activities and sawmill industries. This ratio value is then used to extrapolate wood waste data at the Ciamis Regency level. The wood wastes in the logging activities and sawmill industries were assumed to be the potential supply of firewood. The firewood demand was estimated by considering the consumption of firewood per industry, the number of firewood-using industries, the consumption of firewood per family, and the number of families of the Ciamis residents.

1. The ratio of wood waste to roundwood
  The ratio of waste to roundwood in the logging activities was calculated using the equation below:
  1 $$R_{{\text{B-L}}} = {\text{VB}}/{\text{VL}},$$
  2 $$R_{{\text{T-L}}} = {\text{VT}}/{\text{VL}},$$
  where *R*_T-L_ is the ratio of branches to merchantable roundwood in %, *R*_T-L_ is the ratio of twigs to merchantable roundwood in %, VB is the volume of branches in m^3^, VT is the volume of twigs in m^3^, and VL is the merchantable roundwood volume in m^3^. The ratio of wood waste to roundwood in the sawmill industries was calculated as follows:
  3 $$R_{{\text{S-L}}} = {\text{vs}}./{\text{VL}},$$
  4 $$R_{{\text{Sd-L}}} = {\text{VSd}}/{\text{VL}},$$
  where *R*_S-L_ is the ratio of slabs to roundwood in %, *R*_Sd-L_ is the ratio of sawdust to roundwood in %, and VL is the volume of roundwood that is processed as the input in the sawmill industries.
  There are several units commonly used by timber business actors to calculate the volume of logroundwoods and residues. These units were converted into m^3^ through key informants’ interviews and validated through direct measurements (see Appendix Table 9).
2. Estimation of firewood supply
  Subsequently, the percentage of waste to roundwoods was used to estimate the potential and consumption of firewood at the Ciamis Regency level. The potential for firewood from logging residue was determined by calculating the annual roundwood production value in 2020 [46]. The estimation of firewood from sawmill waste was determined based on the average value of the roundwood volume processed by the sawmill industry and the number of sawmill units in Ciamis Regency considering the *R*_S-L_ and *R*_Sd-L_ values. The total number of sawmill industry units is based on the data from [33]. These potential residues were calculated using the equations below:
  5 $${\text{PLR}}_{{\text{B}}} = {\text{VL}} \times R_{{\text{B-L}}} ,\;{\text{and}}\;{\text{PLR}}_{{\text{T}}} = {\text{VL}} \times R_{{\text{T-L}}} ,$$
  6 $${\text{PSRS}} = {\text{TSm}} \times {\text{aVL}} \times R_{{\text{S-L}}} ,$$
  7 $${\text{PSRSd}} = {\text{TSm}} \times {\text{aVL}} \times R_{{\text{Sd-L}}} ,$$
  where PLR_B_ and PLR_T_ are the potentials of the logging residues of branches and twigs, respectively, in m^3^; VL is the volume of merchantable roundwood, in m^3^; PSRS and PRSd are the potencies of the sawmill residues of slabs and sawdust, respectively, in m^3^; TSm is the total number of sawmill industries (unit); and aVL is the average annual roundwood volume per sawmill in m^3^/unit/year.
3. Estimation of firewood demand
  Firewood demand was estimated by upscaling the firewood consumption of the residents and industries at the Ciamis Regency level. The firewood demand of the residents was estimated as follows:
  8 $${\text{TFwCf}} = P \times {\text{FFw}} \times {\text{FwCf}},$$
  where TFwCf is the total annual demand for firewood for residents in m^3^, *P* is the family number of Ciamis residents, FFw is the percentage of families that use firewood, and FwCf is the average firewood consumption per family in m^3^/year. The population and family numbers in 2020 were estimated based on the population analysis of the Ciamis Regency between 2015 and 2025 [47]. Primary data calculations yielded the percentage of families who used firewood and the average firewood consumption per family.
  The firewood demand for industries (TFwCI) was estimated per industry type based on the sum of production capacity (PC) and the value of firewood consumption per tonne of product or unit product (FwCp) using the equation below:
  9 $${\text{TFwCI}} = \sum\nolimits_{(i = 1)}^{N} {({\text{PC}}_{i} \times {\text{FwCp}}_{i} )} ,$$
  where PCi is the annual production capacity of industry type *i* (*i* = 1,…, *N*) in ton or product units (pieces or chickens) and FwCpi is the firewood consumption per ton or unit products of type *i* in m^3^/ton (or m^3^/1000 chickens or m^3^/1000 bricks). Industry type and production capacity are based on 2020 data from [33], while FwCp is based on the primary data processing.

## Results

### The general condition of SSPF and major tree species

The SSPF in Ciamis District, as well as in other parts of Indonesia, is typically managed traditionally. Planting, maintenance, and harvesting are carried out without using modern technological inputs. Landowners, especially family heads and spouses, are the decision-makers in management practices. The cropping pattern developed in the study area was a mixture of different tree species without specific spacing. Tree monoculture cropping patterns were also discovered when certain species were planted in a regular arrangement. In addition, some people intercropped understory crops, such as cardamom, banana, and cassava, with tree crops in mixed and monoculture patterns.

Timber tree species have their characteristics at all three study sites, with sengon (*Paraserianthes falcataria*), mahogany (*Swietenia macrophylla*), and teak (*Tectona grandis*) being dominant in the Banjaranyar and Ciamis Subdistricts (see Additional file 1: Fig. S2). These species are cultivated by farmers because they are commercial species in high demand by local and regional industries. Mahogany and teak are slow-growing wood species used for carpentry, furniture, and handicrafts. In addition, sengon is a fast-growing tree that is generally used for veneer, bare core, plywood, carpentry, furniture, and formwork boards in local industries and is rarely exported.

**Fig. 2:**
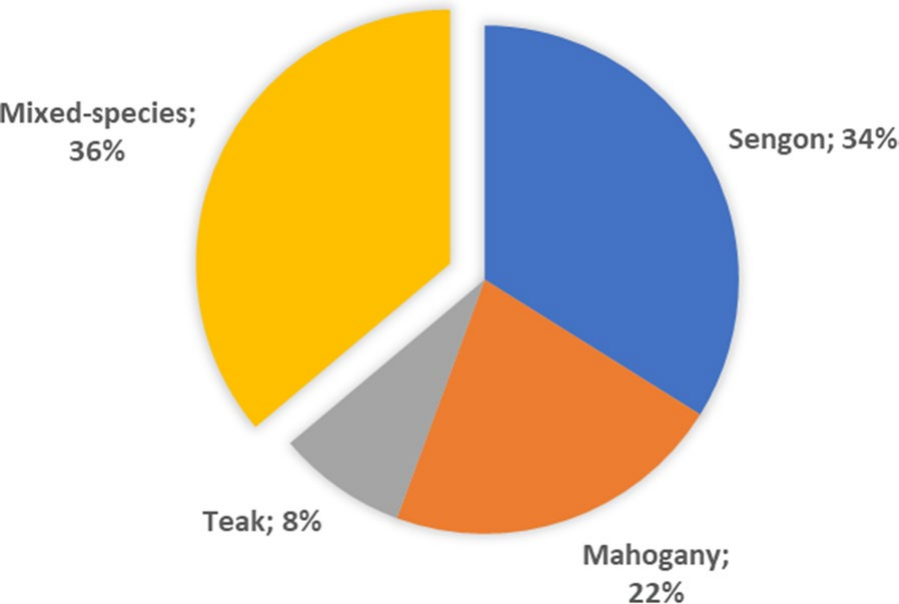
Major tree species logged from the small-scale privately owned forest in Ciamis Regency

Furthermore, fast-*growing* species such as Umbrella Tree (*Maesopsis eminii*) and Ganitri Tree (*Elaeocarpus ganitrus*) were common in Sukamantri District and were agro-climatically suitable for planting in the highlands of the district and its surroundings. The umbrella and ganitri wood were used for carpentry, cast boards, pallet boards, and packaging. The dominance of the sengon, mahogany, and teak in the SSPF of Ciamis District was in line with those observed during logging activities. The volume of roundwoods results measured in 68 logging cases showed that the sengon species occupied the highest share, followed by mahogany and teak, with values of 34%, 22%, and 8%, respectively (Fig. 2). In addition, the remaining 36% was a mixture of lesser-known tree species, which included tisuk (*Hibiscus macrophyllus*), umbrella, ganitri, and gmelina (*Gmelina arborea* Roxb.). Fruit tree species such as dukuh (*Lansium domesticum*), rambutan (*Nephelium lappaceum*), and coconut (*Cocos nucifera*) were also felled when they were no longer productive in some cases.

### Chain of production

Landowners typically sell their trees when there is an urgent need to use a selective logging system. Some farmers who want to replace their trees with new species are expected to use a clear-cutting system to sell all the existing trees. The agreement between the landowner and the timber buyer is sometimes used to determine the clear cutting or selective logging system. The ability to estimate the volume of roundwoods from standing trees was also used as critical for timber buyers in tree transactions.

Timber buyers were assisted during the logging process by loggers and logger assistants who were full-time employees or nonbinding partners. In addition, loggers were chainsaw operators, and their assistants were assigned to measure and transport roundwoods. In addition, a logger also determines the length of the roundwood cuts and sorting of the roundwood sections, such as curved sections, pests, and diseases, and the minimum diameter of the roundwoods for each species to optimize yields during the sawing process. A logger’s or chainsaw operator’s productivity ranges from 8 to 10 m^3^/day for a roundwood length of 1–1.3 m to 12–15 m^3^/day for a roundwood length of 2–3 m.

After buyers and small wood collectors, the next link is the sawmill industry. Roundwoods were obtained through one or a combination of the following methods: (i) buying directly from the sawmill through owners and wood buyers, (ii) obtaining roundwood supply from partner timber buyers, and (iii) providing sawmill services without purchasing wood, which was used approximately by 79%, 43%, and 86%, respectively. Buying roundwoods from farmers and providing sawmill services from others was the most common combination of roundwood sources, which was 36%. Some sawmill owners (29%) also combined all 3 types of roundwood sources and, in a few cases, specialized in sawmill services.

Sawmill owners who bought roundwoods directly from farmers were involved in two production chains, namely, timber buyers and sawmill owners. This is because they are still in partnership with other timber buyers and can directly meet the farmers for timber since most of the owners started their business careers as logger operators. In addition, sawmill industries also collaborated with the previous link, where owners can provide capital loans to timber buyers during the tree-purchase process. Sawmill owners who received roundwoods from partners benefited from less speculation because the roundwood volume was calculated from felled roundwoods rather than standing trees, which gave a greater measurement accuracy. Some industries provide services from roundwood owners without purchasing roundwoods, and the fee is determined by the type of roundwood, which costs IDR200,000, IDR250,000, and IDR30,000 per m^3^ of roundwood for sengon and other mixed species for mahogany and teak, respectively.

Certain qualified roundwoods were sold outside of the Ciamis area, in addition to the local sawmill industry. Sengon roundwoods with a diameter of more than 18 cm were generally sent to the medium/large scale veneer industry around the area. Teak roundwoods were also sold to large wholesalers in areas such as Jepara and Tegal, Central Java Province. In Ciamis Regency, the large wholesalers generally collaborated with brokers as partners. In addition, another product supplied to Surabaya, East Java Province, included sawn timber of mahogany and other mixed species.

**Fig. 3:**
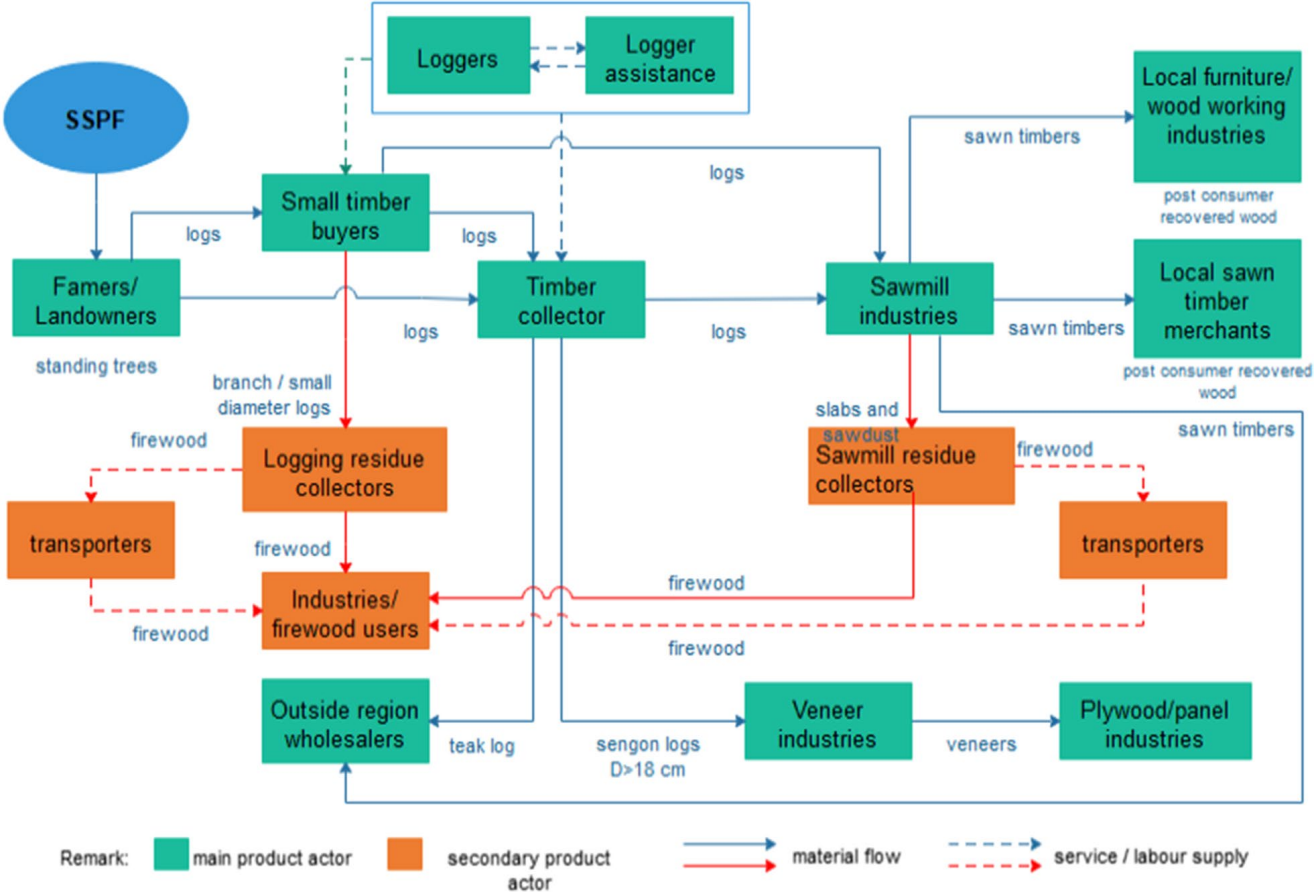
The wood-based production chain of small-scale privately owned forest in Ciamis Regency

Secondary products from the SSPF that become a source of bioenergy included dead branches or twigs from stands, residues from logging, and wood processing activities. Dead branches and twigs from stands, together with twigs from logging waste, are untraded sources of bioenergy for households, while logging residues larger than 5 cm in diameter and wood processing residues were traded for industrial use. Secondary product chains in the wooden product manufacturing process include logging and sawmill residue buyers, middlemen or transporters, and industries that use firewood as an energy source (Fig. 3).

### Wood material flows

People who live close to the SSPF usually pick up dead branches or twigs that have fallen from trees or midribs and flower stalks from palm species. This dead woody biomass can be picked up by anyone without asking the landowners for permission. The biomass from this stand was allocated for a household energy source, particularly for cooking. When logging activities are close to settlements, the community comes to collect twigs for household firewood because they are usually taken up freely.

**Fig. 4:**
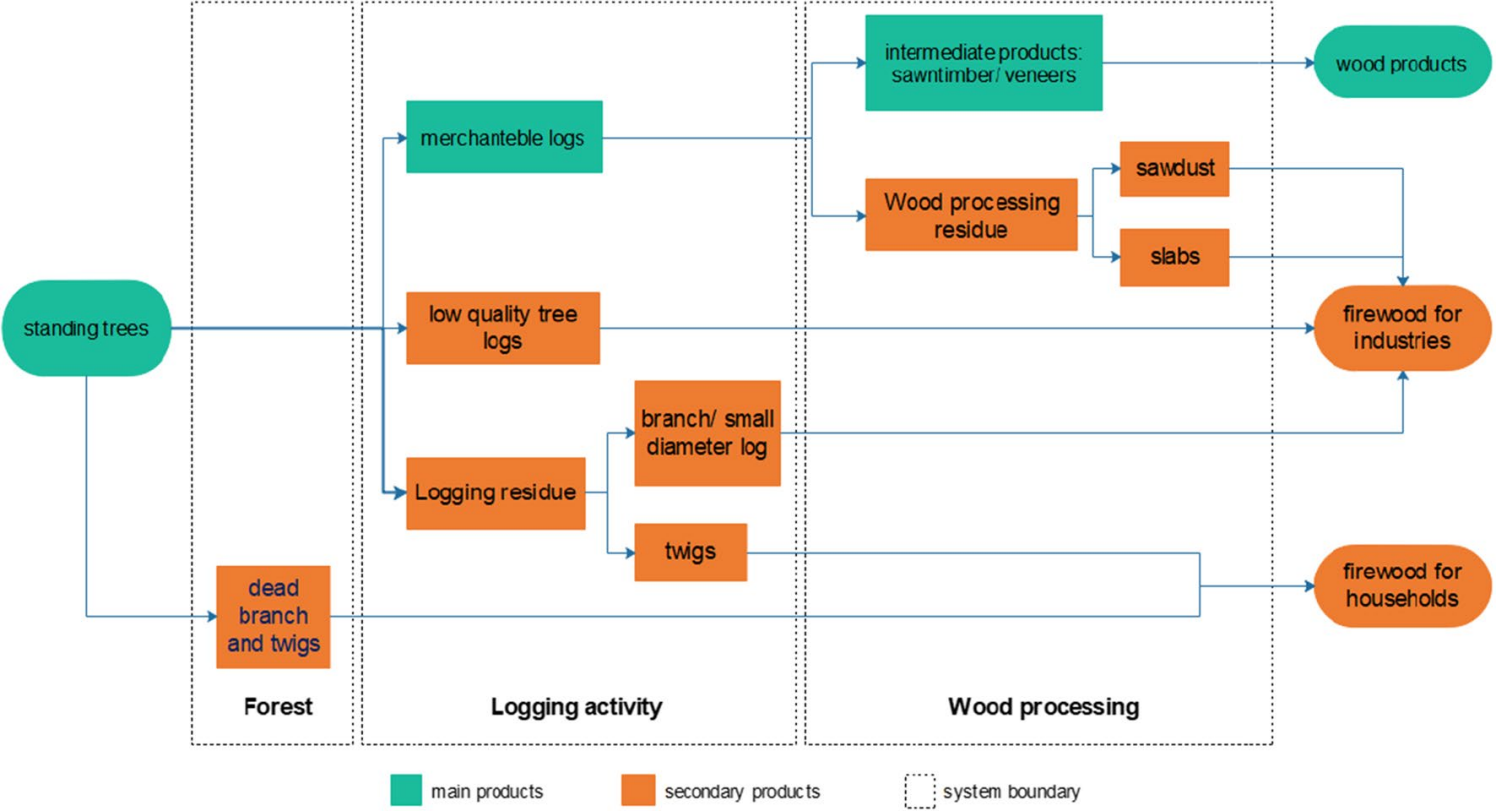
Main and secondary wood material flow derived from the small-scale privately owned forest in Ciamis Regency

Logging waste can be separated into twigs with a diameter of less than 5 cm and branches or a small diameter of 5–10 cm roundwoods. Logging waste in the form of branches or small-diameter roundwoods was a source of firewood that was usually traded for use in industries (Fig. 4). Branches or small-diameter roundwoods are cut into lengths of 60–70 cm and arranged along the haul road. This branch’s length becomes a specific unit measure used to calculate the volume of firewood, namely, 1 *patok* measuring 0.7 m^3^ (1 m × 1 m × 0.7 m). The sales chain can include transporters or middlemen and sell it to end-users; however, waste is not usually purchased by timber buyers. The far distance of the logging locations from road access makes the operational costs for cutting and transporting wood to the roadside not proportional to the selling price. In addition, several firewood seller specialists took advantage of this opportunity by risking transportation costs and purchasing branch waste and small-diameter roundwoods from loggers.

In addition, firewood seller specialists occasionally buy standing trees that are only used for firewood, particularly for low-quality trees that were not classified as merchantable roundwoods. Trees designated entirely for firewood include pests and disease-infected trees, small-diameter saplings, usually in the case of mahogany stands clear-cutting, unproductive fruit-producing trees with roundwoods that cannot be processed as sawn wood, such as rambutan, dukuh, or species known specifically for firewood such as calliandra (*Calliandra calothyrsus*).

Local sawmill industries produce sawn boards and waste from processed roundwoods. Sawn boards came in a variety of shapes and sizes, including boards, planks, narrow boards, strips, scantlings, short sawn timber (less than 1 m in length), battens, ribs, and squares. These intermediate products were sold to local sawn timber merchants or building supply stores. Furthermore, local furniture industries or woodworking industries also obtain sawn board raw materials from sawn timber merchants or directly from sawmills (Fig. 3).

The sawmill industries generated waste in the form of slabs and sawdust, which were also sources of bioenergy for user industries. Many industries use slabs, such as the brick and tofu industries, while others, such as coconut sugar industries, use sawdust. Industrial users preferred mahogany slabs because they have a high calorific value and are durable in the combustion process. Although softwood slabs were relatively abundant and cheaper, industries did not use the slabs as their energy source.

The compatibility of a wooden branch with a length of 60–70 cm and the shape of the stove was also an advantage. Heating stoves in the chicken farming industry usually set the stove for the 60–70 cm length of firewood. Entrepreneurs need to cut branches when the size is not appropriate. Similarly, the shape of the furnace is adjusted to a branch size of 60–70 cm for crackers and sale factories. In addition, certain industries prefer to use slabs because the furnace design allows slab lengths greater than 1.5 m to be adjusted. In the brick industry, several entrepreneurs in Banjaranyar Subdistrict usually combined both branches, which were more durable, and the longer slabs to reach the center of the burnt brick arrangement.

### Potential and consumption of wood energy

1. Firewood potential from logging activities
  The average volume of merchantable roundwoods in each logging activity was 8.9 m^3^. The average ratios of branches and twigs to roundwoods were 104.7% and 54% for *R*_B-L_ and *R*_T-L_, respectively, for a total of 158.7% of the merchantable roundwood volume (Table 3). *R*_B-L_ and* R*_T-L_ varied depending on the branching characteristics of each tree species. Certain species, such as mahogany and sengon, have sympodial branches with a broad canopy, which causes a significant amount of branch and twig waste. In addition, when harvesting mahogany species, a large number of mahogany saplings were occasionally cut during clear cutting. This abundant mahogany sapling was allocated as firewood because it was too small to be a merchantable roundwood. In addition, the ratio of logging waste was low in the umbrella and ganitri because the species have relatively few branches (Table 4).

**Table 3:** Descriptive statistics of the waste to roundwood ratio and firewood to roundwood by logging activities

	Volume of roundwood (m^3^)	Volume of branch (m^3^)	Volume of twig (m^3^)	Waste to roundwood ratio		
				*R*_B-L_ (%)	*R*_T-L_ (%)	Firewood to roundwood (%)
Mean	8.9	5.8	3.2	104.7	54.0	158.7
Minimum	1.0	0.0	0.0	0.0	0.0	32.9
Maximum	74.0	40.6	15.0	800.0	200.0	1000.0
Standard deviation	11.4	6.3	2.9	138.1	49.1	172.0

**Table 4:** Statistics of the waste to roundwood ratio by tree species

Species	Volume of merchantable roundwood (m^3^)	Volume of branch (m^3^)	Volume of twig (m^3^)	Waste to roundwood ratio		
				*R*_B-L_ (%)	*R*_T-L_ (%)	Firewood to roundwood (%)
Sengon, Mean	4.1	2.3	1.2	65.7	47.2	119.7
Minimum	1.0	0.0	0.0	0.0	0.0	12.5
Maximum	21.0	14.0	4.0	333.3	200.0	466.7
Standard deviation	4.4	2.6	1.0	74.9	42.0	104.1
Mahogany, Mean	3.1	2.7	1.6	87.8	58.3	156.6
Minimum	0.5	0.4	0.0	23.3	0.0	46.7
Maximum	12.0	11.2	9.0	280.0	250.0	350.0
Standard deviation	2.4	2.6	1.9	59.3	50.0	88.6
Teak, Mean	4.1	2.1	1.9	51.6	38.1	93.1
Minimum	1.0	0.4	0.3	26.3	17.5	46.7
Maximum	12.0	6.0	6.0	120.0	92.3	183.8
Standard deviation	3.5	1.8	2.0	30.0	24.7	51.7
Mixed species, Mean	3.3	2.5	1.1	82.9	67.2	165.5
Minimum	0.5	0.3	0.1	14.0	7.0	21.0
Maximum	50.0	12.0	8.4	400.0	200.0	500.0
Standard deviation	7.8	4.3	1.5	84.0	56.6	133.3
2. Firewood potential from sawmill industries
  The sawmill industry’s average yield per unit was 66.6%, with a range of 55–78%. One average sawmill unit processes 1468.3 m^3^ roundwood/year and generates 999.4 m^3^ and 274.5 m^3^ of slabs and sawdust wastes per year, respectively (Table 5). The roundwood characteristics of the dominant tree species significantly determined the yield variation. Some sawmill units receive a variety of mixed species of trees from the nearby SPPF area. Other sawmills specialize in certain species, such as sengon or mahogany, particularly in the Ciamis and Banjaranyar areas of the SSPF, where both species are abundant.

**Table 5:** Sawn–timber yields and residues per sawmill industry unit

	Mean	Minimum	Maximum	Standard deviation
Sawed roundwood volume (m^3^/year)	1468.3	360.0	5184.0	1383.8
Yields	66.6%	55.0%	78.3%	7.3%
Residues:				
• Slabs (m^3^/year)	999.4	360.0	2,016.0	492.0
• Sawdust (m^3^/year)	274.5	39.6	950.4	218.0
*R*_S-L_	99.3%	26.7%	200.0%	57.5%
*R*_Sd-L_	22.6%	5.5%	37.7%	10.0%
  Sawmill waste such as sideboards or slabs and sawdust varies based on the species of the roundwood that is sawed. High yields of sawmills almost certainly produce low residues; in addition, sengon and ganitri have relatively high yields, reaching more than 80% (Table 6). Sengon sawn timber has a high yield because it allows for a round shape at the corners of the sawn timber, yielding a thin sideboard. This is related to sengon sawn timber, which is considered low quality and is used for formwork boards or packaging; therefore, high-quality sawn boards are not needed. In addition, the relatively straight and cylindrical shape of the log contributes to the high yield of ganitri sawn timber.

**Table 6:** Descriptive statistics of sawn–timber yields by tree species

Timber species	Yields			
	Mean (%)	Minimum (%)	Maximum (%)	Standard deviation (%)
Sengon	82.3	70.0	95.0	10.3
Mahogany	56.8	30.0	80.0	14.0
Umbrella	65.0	50.0	75.0	10.0
Ganitri	83.3	80.0	90.0	5.8
Mixed-timber species	61.8	50.0	75.0	8.5
All	69.8	56.0	83.0	9.7
3. Firewood consumption at the household level
  Fuel gas and firewood are the primary energy sources for the daily needs of the Ciamis Regency’s residents. The majority of people (55.7%) combine both, while others (38.6%) rely solely on fuel gas, and only a small percentage (5.7%) depend on firewood (Fig. 5). This showed that approximately 61.4% of respondents used only firewood and in combination with gas. Banjaranyar Subdistrict appears to have the highest percentage of firewood use when combined with fuel gas and used solely with firewood, followed by Ciamis and Sukamantri Subdistrict. The survey results showed that people prefer to use gas because it is practical and more convenient to use; however, those who use firewood believe that the supply is abundant, easy to obtain, and relatively cheap (Fig. 6).

**Fig. 5:**
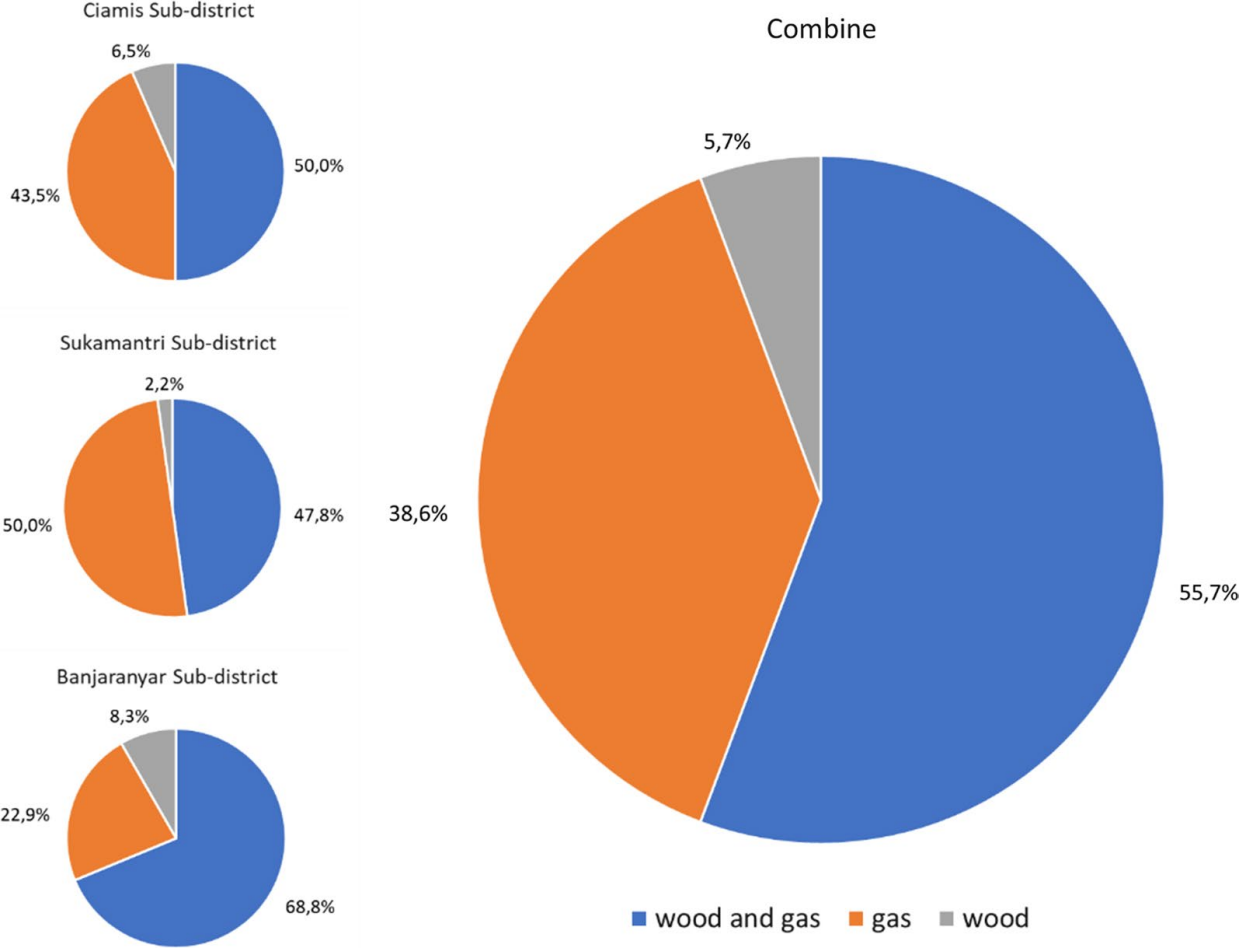
Composition of household energy sources in three subdistricts and Ciamis Regency

**Fig. 6:**
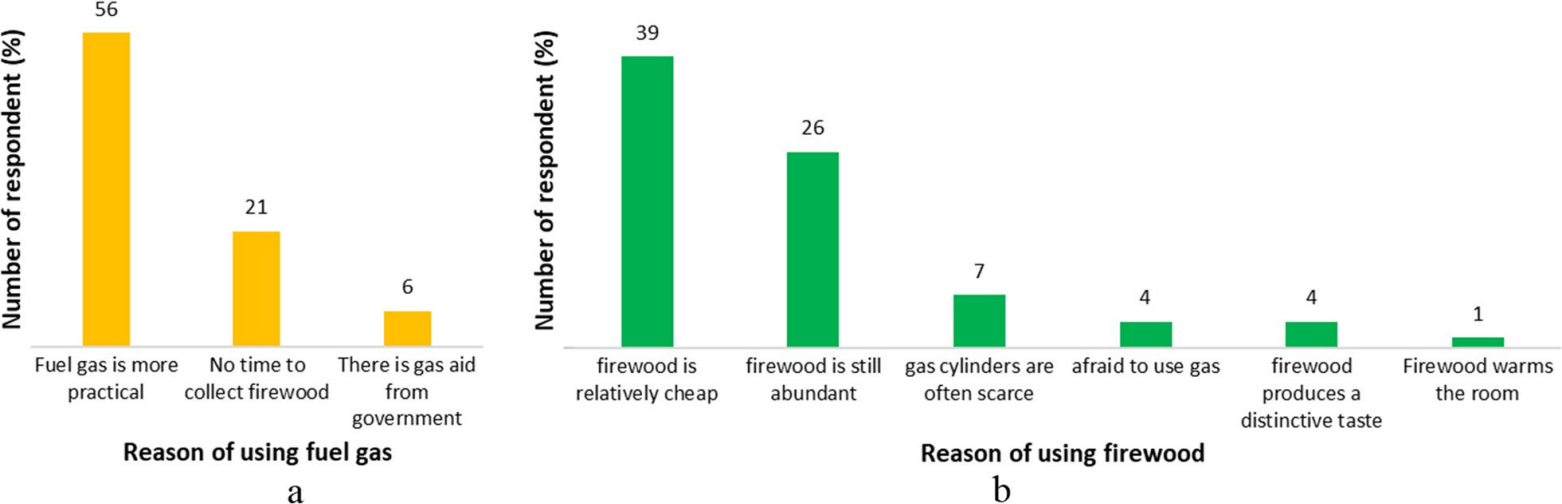
The rationale for using fuel gas (**a**) in contrast to firewood (**b**)
  The average firewood pick up was 10.4 m^3^/year per family, and the extraction significantly ranged from 0.4 to 48 m^3^/year, as shown in Table 7. The annual use of firewood was less than 1 m^3^ per family, where it was only used occasionally and more routinely in the use of gas. The average household consumption of firewood, which depends on family needs, was the highest in Banjaranyar Subdistrict and lowest in Sukamantri Subdistrict. In addition, the relatively low volume of firewood extraction in the Sukamantri Subdistrict was assumed to be related to the weather in the area, which has high rainfall, thereby reducing the opportunity to collect firewood. Therefore, the use of gas was higher even though firewood was significantly abundant due to the large potential of the SSPF stands.

**Table 7:** Firewood from forest for household consumption

Region	Firewood taken per household (m^3^/year)			
	Mean	Minimum	Maximum	Standard deviation
Ciamis	10.1	0.8	48.0	12.1
Banjaranyar	12.3	0.7	33.6	10.7
Sukamantri	7.8	0.4	24.0	7.2
Combine	10.4	0.4	48.0	10.4

This figure was calculated from firewood-user respondents, totaling 86 of 140 households (61.4% of total respondents)
  The collection of firewood was usually carried out by the head or wife of the household. People usually do not allocate time specifically to collecting firewood but rather pick it up during their activities in the garden. Therefore, a wife who often works in the garden assists in the collection and transportation of firewood, which was traditionally tied and carried by hand manually. The weight of one bundle carried by a woman ranged from 4 to 13 kg, with an average of 7.4 kg, according to the measurement of the sample of firewood ties. In addition, one bundle of firewood carried by a man weights between 5 and 30 kg, with an average of 18 kg. When a husband and wife garden together, they can divide labor, where the wife collects the firewood and the husband does the hauling.
  The rate of obtaining firewood depends on the volume required and the opportunities available to each family. Communities whose main activity is gardening can collect firewood every day, even though the number is only one bunch, while others collect firewood weekly or two to three times a month. As previously stated, twigs from logging residue are also used as a source of firewood in the community.
4. Firewood consumption at the industrial level
  In Ciamis Regency, the industries that use firewood are grouped into micro and medium industries and are classified into agro-industry groups such as copra, tofu, palm/coconut sugar industries, and other industry groups, such as the brick industry [33]. The annual production of micro- and small-scale industries of food types such as copra, tofu, snacks, and palm/coconut sugar is 41 tons/year per industrial unit and 19,000 chickens/year, as well as 106,000 bricks per business unit. Firewood consumption ranges between 1.9 and 13 m^3^ per ton of product, 4 m^3^/1000 chicken, and 0.9 m^3^/1000 bricks. The average transaction value for the volume of firewood consumed by the industry was IDR13.4 million per micro- and small-scale business unit and IDR540 million per medium-scale business unit (Table 8).

**Table 8:** Various industries using firewood for energy sources and firewood consumption

Scale of industry	Type of industry	Firewood transaction value (IDR/year)	Average annual production per industrial unit		Firewood consumption	
			Mean	Unit	Mean	Unit
Micro and small	Copra	8,064,000.0	81	Ton	1.9	m^3^/ton
	Tofu	35,000,000.0	53	Ton	9.1	m^3^/ton
	Snack	7,447,000.0	26	Ton	11.2	m^3^/ton
	Aren/coconut sugar	13,350,000.0	3	Ton	13.7	m^3^/ton
	Poultry farm	4,300,000.0	19,000	Chicken	4.0	m^3^/1000 chickens
	Brick	11,800,000.0	106,000	Piece	0.9	m^3^/1000 pieces
Medium	Kerupuk industry	540,000,000.0	430	Ton	10.6	m^3^/ton
  Branch or small-diameter roundwoods from logging residue are the most commonly used type of firewood by industry, followed by slabs from sawmill residue (Fig. 7). The branches of the most popular tree species include mahogany, teak, calliandra, and various mixed species of hardwood. Despite the abundance of sengon logging waste, not all industries receive the type of specialization, except as a mixture. Mahogany or other hardwoods are also among the popular types of firewood in the form of slabs. Because the sawing yield of sengon is relatively high (Table 7), there are few slabs of this species available. Sengon wood slabs are also rarely used as firewood due to their relatively soft characteristics.

**Fig. 7:**
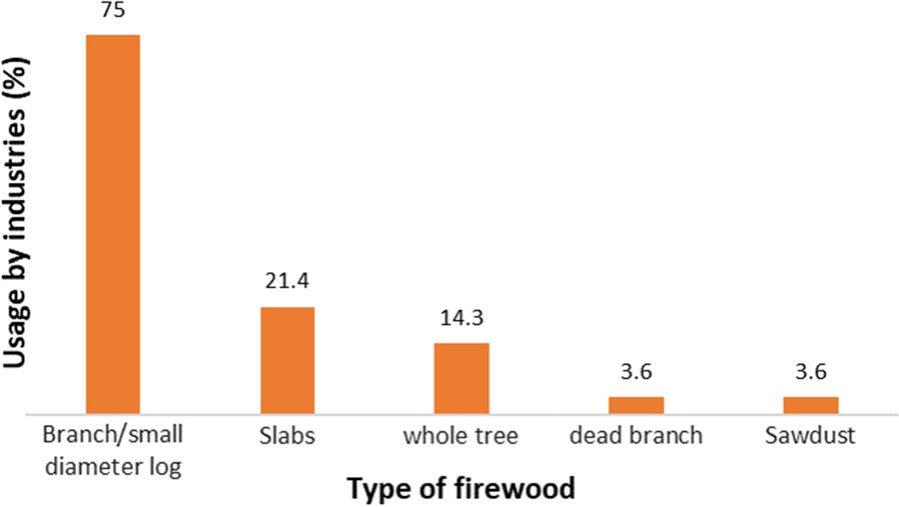
The proportion of different types of firewood used by industries
5. Firewood supply and demand balance at Ciamis Regency
  The total annual demand for household firewood in Ciamis Regency was estimated to be 2.48 million m^3^. This value was obtained from the 2020 population of 1,201,685 people with 387,640 families [47], assuming a firewood-user percentage of 61.4% (Fig. 5) and household consumption of 10.4 m^3^ per year (Table 7). In addition, firewood demand for industries was estimated to be 6.03 million m^3^ per year, while aren/coconut sugar industries required the most, with a value of 4.98 million m^3^ per year. This value was mainly obtained from five small- and medium-scale industrial units with a combined production capacity of 364,493 tons/year [33] and a firewood demand of 13.7 m^3^/ton of product. The poultry farm industries have the next highest demand, with a capacity of 105 million chickens/year and a need for firewood of 4 m^3^/1000 chickens, which requires 0.42 million m^3^/year.
  Based on the data on the 145,469.9 m^3^ roundwood production in 2020 [48], the estimated supply of firewood from logging waste was 190,330.1 m^3^ (Fig. 8). The potential for firewood in the form of branches and twigs was 109,263.6 m^3^ for branches and 81,066.5 m^3^ for twigs. In addition, the potential for firewood from sawing waste was estimated to be 854,282.2 m^3^, where slabs have 695,816.3 m^3^ and sawdust has 158,456.9 m^3^. With a different approach, where the sawmill waste was calculated based on the total volume of roundwoods after calculating the *R*_S-L_ and *R*_Sd-L_ values, the total value of firewood in 2020 is 367,767.7 m^3^. This firewood figure was estimated to be 72% of the total wood biomass of roundwood flow from the SSPF in the Ciamis Regency.

**Fig. 8:**
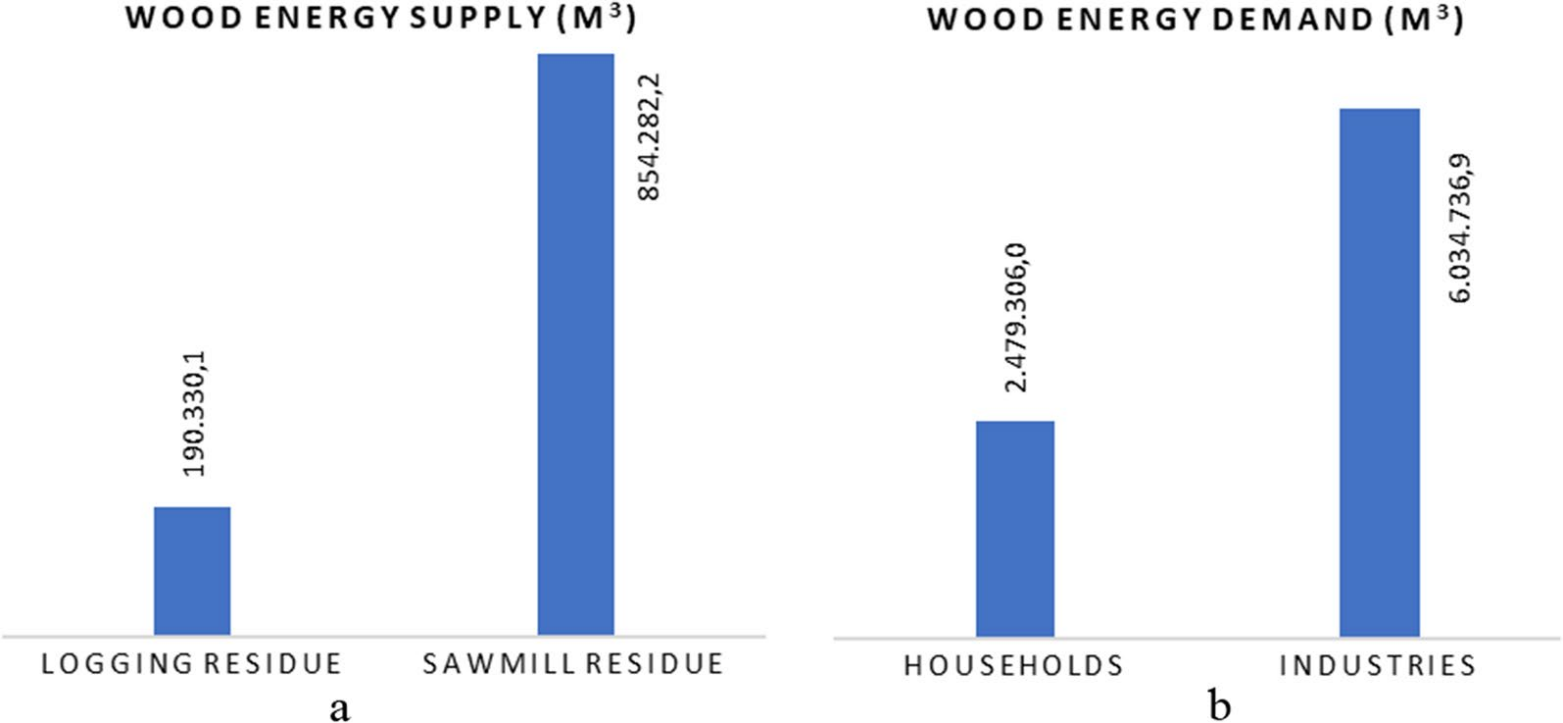
Estimation of the annual supply (**a**) and demand (**b**) for wood energy in Ciamis Regency

## Discussion

The commercialization of firewood is inseparable from the main products’ production system because it is a byproduct sourced from the SSPF in Ciamis. Sawn timber, bare core, plywood, veneer, and blockboard are the main products of Ciamis’ timber-based enterprises [46], which include micro, small, and medium-sized businesses. In 2019, the Ciamis forest product industry had 477 units with a total investment value of IDR54.1 billion [33]. The major tree species developed in the study area respond to market demand, which is shown in the dominant species of mahogany, teak, and sengon. A previous study [42] reported that the roundwood production of the 3 species was 128,937 m^3^, or 66.3% of the total roundwood production in Ciamis Regency in 2019, and are commercial wood species with high demand in local, regional, and national markets.

The dominant species in the Ciamis District SSPF influence bioenergy use; therefore, the abundance of firewood sources from stands, logging waste, and wood processing industry waste is directly related to the abundance of these tree species. Although the use of wood biomass as firewood necessitates certain qualifications, firewood users make significant use of the available resources. Based on this study, the most popular types of firewood in the industry are mahogany branches, slabs, and other hardwood species, which are also abundant in the forest. According to [49], people in Magelang Regency and Central Java used mahogany as firewood because it has a high caloric value and dominates the privately owned forest. It was also stated that [50] people on Java Island, namely, Banjarnegara, Sukabumi, and Banten Regencies, used calliandra and gliricidia (*Gliricidia sepium*) firewood because the species were abundant in these areas.

Ciamis residents generally recognize that hardwood is a good material to use for firewood based on their knowledge and experience. People are aware that mahogany, teak, and other mixed-tree species, such as puspa (*Schima walichii*), dukuh, and rambutan, are typically hardwoods that produce a long-lasting flame when used as firewood. Calliandra is a woody shrub considered a weed in commercial tree cultivation. This species is also known for producing high-quality firewood, which is easier to ignite, even when it is not completely dry. This is in line with a study by Ramos et al. [51], which stated that the preference for firewood species by traditional Brazilian communities is related to the high physical quality of wood and is indicated by the high value of the Fuel Value Index (FVI). The local knowledge of Ciamis residents seems to confirm the basic theory of the relationship between the physical characteristics of the wood and the caloric value. The calorific value of the fuel determines its quality, which is the amount of heat required to raise the temperature of one pound of water by 255.928 Kelvin [52]. In addition, the specific gravity of wood is related to its high calorific value, where the specific gravity value indicates the amount of lignocellulose in a given volume of wood [52]. The greater the conversion of lignocellulose content into energy, the higher the specific gravity of wood. Hardwood is easily identified as wood with a relatively high specific gravity.

The actors involved in the production chain of the main wood-based products are an integral part of the firewood sales chain as a secondary product. However, actors specifically engaged in the firewood sector interact with those in the main product chain. Observations in the field showed that the firewood business offers a viable source of income for entrepreneurs with relatively low capital compared to the main product. The firewood industry also contributes to the local economic activation of the industrial sector by providing low-cost, renewable energy and increasing the added value of waste. This is in line with the assertions of [16] and [17], which stated that the value of converting waste wood products into wood energy increases income at both the company and community levels.

In addition, the presence of local wisdom in resource allocation is one of the unique characteristics of using wood as an energy source in the Ciamis Regency. The community established a clear distinction between household and industrial firewood, which showed that there is a social consensus, where firewood is a primary need that should be prioritized by both households and industries. Although the primary goal of managing the SSPF is for timber production, firewood as a byproduct is still valued by the community. This local wisdom is in line with a study by Hodges et al. [53], which showed that private forestland owners are generally willing to develop their forestland as an energy source, considering the long-term market and future value.

Another local wisdom on resource sharing in the provision of bioenergy sources for households stated that communities are free to collect firewood such as dead branches and twigs from nearby forestlands without seeking permission from the landowner. Similarly, only small-diameter branches and roundwoods are traded during logging activities, while twigs less than 5 cm in diameter can be freely taken by the local community. This phenomenon is also one of the ways to maintain good relations between loggers and the surrounding community when there are logging activities. Twigs taken freely are compensation for the surrounding community, for logging activities that use local road infrastructure, or cause noise pollution or other negative externalities. This shows that the use of firewood by related communities is part of social capital, which varies based on the geographic, cultural, and social meaning [54].

One of the unique aspects of SSPF resource ownership and access is the sharing of SSPF products in the form of firewood for households. The land status of the SSPF is private property; however, the firewood product allocated to households becomes a common property that can be accessed by a group of people [55]. This phenomenon shows that firewood is an essential item for meeting basic needs, without considering its classification as a byproduct in the SSPF business’s production chain.

The contribution of SSPF to meeting household and industrial energy needs is very important. In 2020, the volume of household and industrial firewood consumption was expected to be 2.48 million m^3^ and 6.03 million m^3^, respectively (Fig. 8). However, the estimated potential for firewood from logging waste and sawing waste in the same year was only 190,330.1 m^3^ and 854,282.2 m^3^, respectively. Total firewood derived from logging and sawmill residues was 72 percent of the total woody biomass flow from Ciamis’ SSPF. This shared value is greater than the reported use of biomass for fuel from the forest sector in Germany by [56], which is only 60%, or 20 million m^3^ of 33 million m^3^ of dry biomass. Similarly, [57] in Brazil and [58] in Ethiopia reported 20–30% and 46.5% forest residue for bioenergy, respectively. In this study, the relatively high residue of wood biomass indicates a low efficiency of wood processing but also supports the supply of renewable energy, which is less expensive and more environmentally friendly than fossil fuel.

The high demand for firewood was because several snack food industries have a relatively high production capacity, which is more than 7 tons of product per year, with the use of 5 m^3^ of firewood per ton of product. In addition, the coconut sugar industry uses firewood at a relatively high intensity (17 m^3^ per ton of product), leading to high demand for firewood at the district level aggregate. The firewood estimation for this industry can be overestimated because the calculation is based on the maximum production capacity data available from the [33]. In addition, the potential for firewood in the Ciamis Regency may be underestimated because due to lax regulations, not all community timber distribution is reported to the Forest Official.

Based on the observations and interviews with key informants from firewood suppliers and industry players, the use of firewood in Ciamis Regency is increasing. This is in line with the growing number of industries in the regency that rely on firewood. Firewood suppliers who previously supplied to the roof tile and brick industries in the Kuningan Regency area have shifted their focus to local industries in the Ciamis region. This is because some medium-sized businesses in Ciamis also have a high demand for firewood. The inequity that occurs in meeting the demand for firewood in the Ciamis industry is currently partially supplied by nearby areas such as Pangandaran Regency and Banjar City. Although the area of Pangandaran Regency is not as large as that of Ciamis Regency, roundwood production in 2020 reached 208,255.6 m^3^ or 43 percent more than roundwood production in Ciamis Regency [48]. This high rate of roundwood production increases the potential for firewood from logging and sawing waste. In addition, the unreported tree harvesting activities conducted by individuals should be other firewood sources that were not covered in the calculation of this research. The cases when loggers buy standing trees entirely for firewood (such as pest and disease-infected trees, trees that cannot be processed for sawn wood) were also not included in the estimation. In addition, some industries also consume a mixed energy source of coal or gas to complement firewood, while the data on the number of industries using mixed energy sources were difficult to obtain.

The high intensity of firewood use as an energy source is related to the abundant supply in the Ciamis Regency and its surroundings, which is available at relatively low prices. This shows the benefits of firewood to the community's economy by serving as both an alternative energy source and generating revenue for the chains involved in firewood transactions. In addition, as the industry develops, the increasing demand for firewood necessitates good forest resource management as its carrying capacity. This needs to be considered because the main challenges in achieving energy self-sufficiency from small-scale forests are related to limited land, capital, and labor [59]. Owing to an increase in the value of small diameter and low-quality tree species [16] or from wood waste, the use of wood biomass as an energy source increases income at the company and community levels [17]. However, a previous study [60] stated that excessive firewood use can put a strain on natural resources. Therefore, the use of forest biomass residues for firewood needs to pay attention to the balance of supply and demand. Some of the efforts that can be made include regulating forest waste harvesting that follows guidelines agreed upon by stakeholders [61], efficiently using firewood [62], and considering the implementation of carbon trading policies [63]. These efforts also need to be adjusted to the local context for effective results.

In the short or medium term, shifting from traditional to the modern use of bioenergy through technology input seems to be a possible option for more efficient energy use. Among the efforts is a stove improvement to obtain a more efficient wood heater. An improved stove has a chance to decrease CO_2_ emissions by a factor of 1.3 [21], while an improved stove ignition technique using top-down ignition of a wood crib can reduce CO_2_ emissions by 73% of the traditional technique [64]. Adding an emission control using a honeycomb catalyst can also reduce 73% of carbon monoxide, 58% of organic gaseous carbon, and 33% of particulate matter [65].

The development of an EPF in SSPF is also another option to overcome the firewood supply–demand gap. The development of EPF can be applied by cultivating short-rotation tree species with a coppice harvesting system. Some tree species, such as *Acacia auriculiformis*, *A. mangium*, and *Calliandra callothyrsus*, can be developed for the EPF due to their high caloric value, fast growth, and ability to grow in a wide range of biophysical conditions of sites [66, 67]. The establishment of EPF in SSPF can contribute to fulfilling the domestic energy demand while supporting governmental targets on renewable energy mix.

## Conclusions

The use of biomass from forests as an energy source still continues and appears to be increasing as fossil fuels are limited and population and industry grow. The case of SSPF in Ciamis Regency could be evidence that bioenergy from smallholder forests contributed to meeting the energy needs of residents and industries. This case study could also be a reference for the traditional use of bioenergy, which may persist in various parts of the world. The traditional use of bioenergy was a foothold in seeing local perspectives to meet global demands related to new and renewable energy. However, a mismatch between the potential supply and the demand for firewood was detected. This is partially due to the high intensity of firewood consumption in local industries at full production capacity, which can put additional strain on natural resources. This indicated that SSPF can be used to support future energy security by ensuring the availability of affordable and renewable bioenergy while considering the sustainability of SSPF resources.

Because it was considered a secondary product, bioenergy from SSPF was frequently overlooked in its management. A high-intensity consumption of firewood with low efficiency can still be at risk of increasing CO_2_ emissions. Therefore, various efforts are needed to increase the efficiency of the use of firewood and to promote sustainable forest management. Multistakeholder and cross-regional collaborations are required to achieve sustainable SSPF management by meeting the needs of wood-based biomaterials as the primary product and bioenergy as a secondary product.

This study ignored the bioenergy business process originating from outside or around the Ciamis Regency area, which may be involved in bioenergy supply and demand in this region. To some extent, this could be a source of bias to estimate bioenergy supply and demand more accurately, considering that the market chain cannot be limited by administrative areas. Unreported logging activities were also one of the potential bioenergy supplies that were not recorded in this study. Therefore, further research on a wider chain beyond the administrative boundaries can increase the accuracy of the bioenergy supply and demand estimation. Research involving spatial analysis at the forest stand level could be an alternative to estimate the potential of forests and the biomass residue they produce. In addition, a survey on the use of firewood involving a larger sample size can increase the validity of the study.

### Supplementary Information

**Additional file 1: Figure S1.** Source of energy from forest biomass, logging residue, and sawmill residue. **Figure S2.** The appearance of small-scale privately owned forests with several dominant tree species in Ciamis Regency.

## Data Availability

The primary datasets used and analyzed during this study are available from the corresponding authors on reasonable request. The secondary data that support the results of this study are available from the Local Government of Ciamis Regency, but restrictions apply to the availability of these data, which were used under license for this study; therefore, they are not publicly available.

## Appendix

See Table 9.

**Table 9:** Conversion of local volume units of firewood

Volume unit	Conversion	Remarks
1 bunch	1/16 m^3^	Volume of branch or twigs
1 *patok*	0.7 m^3^	Volume of branch, small-diameter roundwoods, twigs, slabs
1 wagon	1 m^3^	Volume of branch, small-diameter roundwoods, twigs, slabs
1 ankle	7 m^3^	Volume of branch, small-diameter roundwoods, slabs
1 truck	15 m^3^	Volume of branch, small-diameter roundwoods, slabs
1 coltbak = 1 hiace	3.5 m^3^	Volume of branch, small-diameter roundwoods, slabs
1 coltbak = 1 hiace	80 bag	Volume of sawdust
1 bag	0.066 m^3^	Volume of sawdust

## Abbreviations

SSPF: Small-scale privately owned forest
EPF: Energy plantation forest
*R*_S-L_: Volume ratio of slabs to roundwood
*R*_Sd-L_: Volume ratio of slabs to roundwood
*R*_B-L_: Volume ratio of branches to roundwood
*R*_T-L_: Volume ratio of twigs to roundwood
