# Supplementary material for: Renewable energy from secondary wood products contributes to local green development: the case of small-scale privately owned forests in Ciamis Regency, Indonesia
Source: Energy Sustain Soc. 2023 Feb 14;13(1):4. doi: 10.1186/s13705-023-00383-7 (PMC9926445; doi:10.1186/s13705-023-00383-7)
Supplement: Supplementary file 1 — Additional file 1: Figure S1. Source of energy from forest biomass, logging residue, and sawmill residue. Figure S2. The appearance of small-scale privately owned forests with several dominant tree species in Ciamis Regency. [file 13705_2023_383_MOESM1_ESM.docx]

**Additional file 1**


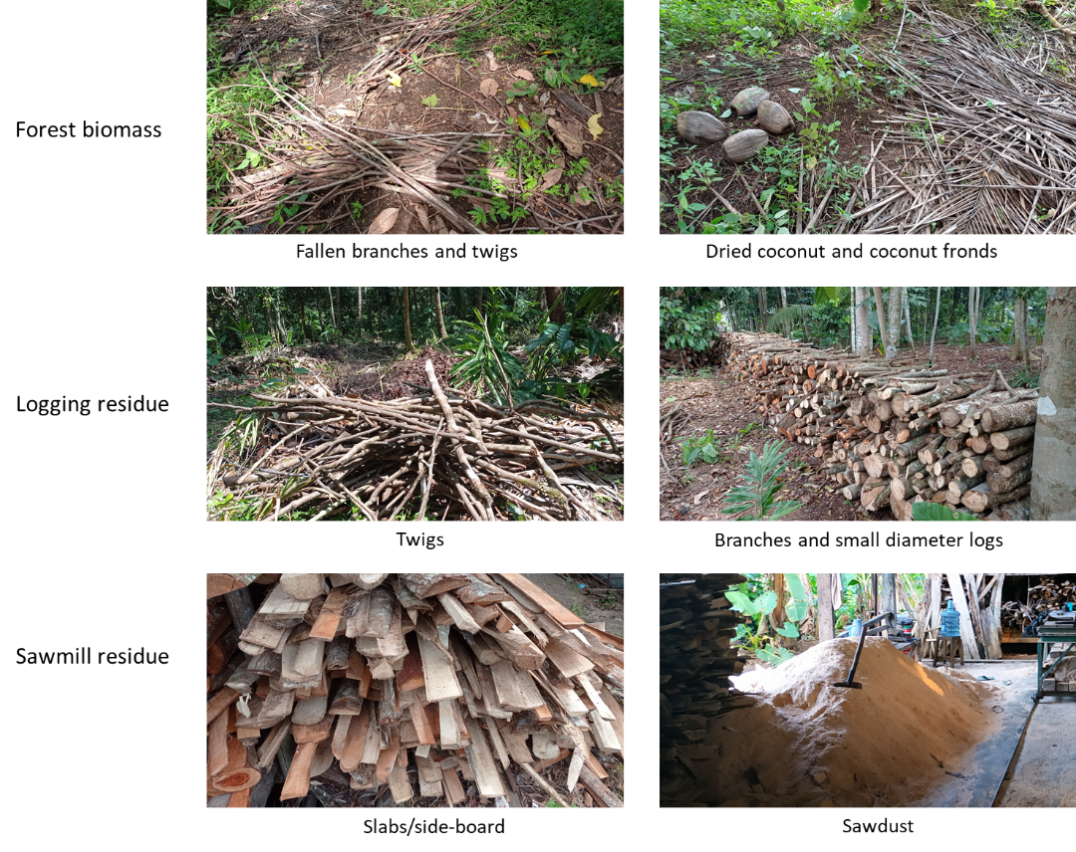


Figure S1. Source of energy from forest biomass, logging residue, and sawmill residue


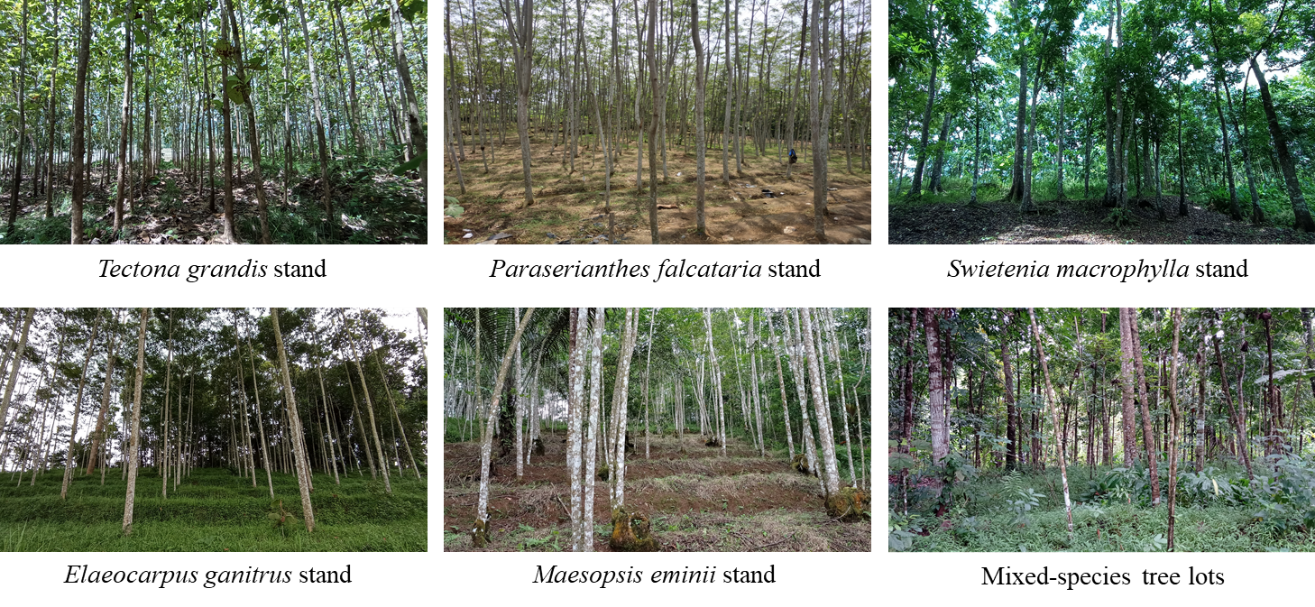


Figure S2. The appearance of Small-scale privately-owned forests with several dominant tree species in Ciamis Regency
